# Pediatric Emergency Medicine Didactics and Simulation (PEMDAS): Outside Medical Emergency Team Response

**DOI:** 10.7759/cureus.80637

**Published:** 2025-03-15

**Authors:** Alexandra P Licona-Freudenstein, Cassandra Koid Jia Shin, Brian Burns, Jennifer Reid, Catherine Nguyen, Sara Fenstermacher, Anita Thomas

**Affiliations:** 1 Pediatric Emergency Medicine, Seattle Children's Hospital, Seattle, USA; 2 Pediatric Emergency Medicine, University of Washington, Seattle, USA; 3 Pediatric Emergency Medicine, Tulane University School of Medicine, Seattle, USA; 4 Pediatrics, Louisiana State University Health Sciences Center, New Orleans, USA

**Keywords:** acute coronary syndrome, anaphylaxis, medical emergency team (met), opioid use disorders, pediatric emergency department, problems during pregnancy, simulation in medical education

## Abstract

The United States’ Emergency Medical Treatment and Active Labor Act applies to individuals who present within hospital grounds. Thus, emergency preparedness is essential outside the hospital and on campus grounds. This technical report describes a novel simulation-based curriculum for pediatric emergency residents, fellows, and attendings responding to medical emergencies outside the hospital but on campus grounds. This curriculum highlights the importance of effectively responding to emergencies on hospital grounds outside the pediatric emergency department. The simulation-based curriculum was conducted at two pediatric emergency departments and involved pediatric emergency medicine fellows and attendings. Simulation participants were prompted to complete a post-simulation survey. The survey results demonstrated that participants felt that this simulation curriculum was relevant to their work and that they felt more confident in their ability to respond to an out-of-hospital emergency after participating in the simulation curriculum.

## Introduction

In 1986, the United States (U.S.) Congress passed the Emergency Medical Treatment and Active Labor Act, which applies to individuals within 250 yards of the hospital, including hospital parking lots, sidewalks, and adjacent medical buildings [1]. In the U.S., those individuals are entitled to a medical screening by a physician or a qualified medical person and stabilization to identify an "emergency medical condition" [1,2]. Regulations also exist outside of the U.S. to ensure access to emergency medical care for all individuals, including those near the hospital [3]. An emergency medical condition is defined as acute symptoms of sufficient severity by which the lack of immediate medical attention could place the health of the patient, or (in case of pregnancy, the life of the fetus), in serious jeopardy, significant impairment to bodily functions, or serious dysfunction of any bodily organ or part [1]. This translates to offering at least a medical screening exam (MSE) to anyone who presents to a hospital in the U.S., regardless of age or pregnancy status, and despite whether it is a specialized hospital, such as a children’s hospital. Thus, pediatric emergency medicine (PEM) physicians who work at such places must be well versed in responding to out-of-hospital adult emergencies in hospital campus areas that may require transfer or initial stabilization and out-of-hospital emergencies of non-hospitalized pediatric patients with limited equipment and resources.

Literature on the systematic approach to responding to medical emergencies in non-hospitalized persons, including those on campus grounds outside the hospital building, is lacking [4]. One retrospective review of rapid response systems events that occurred in non-hospitalized patients outside of the intensive care unit (ICU) and on campus grounds over a 28-month period found that perceived emergencies occurred commonly, and most patients required further evaluation in the ED [5]. Similarly, a retrospective study that examined code blue responses at a large children’s medical center described that 80% of code blue responses occurred in non-hospitalized persons and are more likely to involve adults [6]; hence, highlighting the need for preparedness in responding to such medical emergencies, particularly for those who work in specialized hospitals, such as children's hospitals.

There are no published standards for the composition of team structure for the medical emergency team (MET) that responds to emergencies involving non-hospitalized persons [5]. Therefore, the team structure of the MET varies by institution. However, the overall management of the MET is the same: (1) A physician or qualified medical person performs an MSE to determine whether the individual has an emergency medical condition and (2) whether the individual requires stabilization and treatment within the hospital’s capabilities prior to transfer to an outside facility, if specialty capabilities are required. At many institutions, these responsibilities fall to a code blue team, a rapid response team, the ICU, or the emergency department. At our primary institution, the ED is responsible for incidents on the hospital campus outside of the main physical building (e.g., parking areas, adjacent outpatient clinics), and the ICU or code blue team is in charge of internal incidents within the main hospital building. These response teams are frequently staffed by trainees, either a fellow or resident, and nurses. While simulation cases that address MET responses for hospitalized persons have been published, to our knowledge, simulation cases focused on pediatric emergency personnel responding to hospital campus emergencies of non-hospitalized persons have not [7]. These are low-frequency, high-risk scenarios that PEM physicians, particularly trainees, must respond to. Thus, we aim to use this technical report to simulate effectively performing an MSE and determine triage and disposition for adult, pregnant, and pediatric non-hospitalized persons.

## Technical report

Methods

Case Overview

PEM physicians developed the simulation cases with expertise in curriculum development and simulation. The four case scenarios were based on potential outside medical emergencies of non-hospitalized persons, including an unresponsive adult, a syncopal pregnant person, an adult with chest pain, and a child with anaphylaxis. Each case was written to occur on different hospital grounds. Participants were expected to locate the patients once the MET was activated and assess the patient to determine if the patient required (1) stabilization at the scene with transfer and definitive treatment in the PED, (2) stabilization at the scene or PED and treatment within the capabilities of the PED to ensure a safe transfer to another facility for definitive treatment, or (3) stabilization at the scene and immediate transfer to an outside facility for definitive treatment. The simulation case scenarios were originally carried out with PEM fellows at a single site. Two groups of two to three fellows were assigned to two of the four case scenarios, and the simulations were run simultaneously. Feedback from the original simulations resulted in modifications to the participant structure, in which first- and second-year PEM fellows participated together in all four case scenarios, and second-year PEM fellows helped guide the first-year PEM fellows through the simulation. Additionally, third-year PEM fellows became embedded participants (EPs) and helped facilitate the simulation. Revised simulations were run with PEM fellows, attendings, and pediatric ED ancillary staff at an additional institution as part of their routine education curriculum. No prerequisite knowledge was required, although at the initial institution, a brief orientation to the MET response was given prior to running the simulation based on iterative feedback. These simulations were conducted at the beginning of the academic year, when first-year fellows may not have had knowledge of or experience with a MET.

Participants

PEM fellows were the primary target audience. Other targeted audiences included other PEM providers, ED nurses, respiratory therapists, and ancillary staff who may participate in the MET response at various institutions. We implemented this simulation with 10 participants, including five PEM fellows and five PEM attending physicians, across two training sites over six months. Participants had previous experience with simulation and medical resuscitation. Participants were oriented to the MET equipment and personnel. Each site was provided with the four case scenarios to run through each scenario once.

Personnel

The facilitators included PEM attending physicians, third-year PEM fellows, and nursing leadership. Ideally, the sessions were run with at least two facilitators; however, given resource limitations, the simulations were occasionally run with one facilitator. No high-technology manikins were utilized in the simulation, so no technician was required.

Setting and Equipment

Each case scenario occurred outside the PED, including various hospital clinics, hospital parking lots, and parental respite areas. The locations were designed to be modified depending on where pediatric ED personnel are expected to respond to MET responses at their respective institutions. Equipment carried by the MET varies by institution and may include blood pressure cuffs, pulse oximeters, epinephrine autoinjectors, intranasal naloxone, bag valve masks, and code charting materials. These cases can also be adjusted based on the availability of EPs.

Environmental Preparation

We recommended the facilitators arrive at least 30 minutes before participants' arrival to set up in each location outside the emergency department. High- or low-fidelity manikins or EPs may be used depending on institutional resources. All participants were provided with the patient's location outside the PED and a short description of why the MET was called.

Scenario Template

Facilitator guides were developed for the four case scenarios (see Appendix A) following a MET activation for facilitators and EPs. Each guide outlines the primary learning objectives, critical actions, and decision points.

Prebriefing

We recommended that all participating sites conduct a pre-brief: an introduction to the simulation, discussion of the simulation learning contract and expectation setting, an orientation to MET, and MET supplies and personnel. Facilitators were given the option to use a low- or high-fidelity mannequin. If facilitators decided to use a mannequin, participants had to introduce the mannequin’s capabilities to the facilitators or simulation technologists during the pre-brief. We advised that this pre-brief be conducted outside of the simulated space. In this way, distraction is minimized, allowing less experienced learners to gain background knowledge on the MET response. We advised allotting 10 minutes at the minimum for pre-briefing.

Case Scenarios

Case 1 (opioid overdose): Case 1 presented participants with an unarousable parent found in an outpatient clinic waiting room. The learning objectives of this case included locating the patient outside of the emergency room, recognizing that the patient has altered mental status with concern for opioid overdose, verbalizing a differential diagnosis for altered mental status, obtaining vital signs and a physical exam, as well as performing interventions including a point of care serum glucose and an electrocardiogram (ECG) by asking the outpatient clinic for those resources. Key steps in the simulation included administering naloxone and providing airway support as needed, recommending evaluation in their respective ED for stabilization or to call 911 if transfer to an outside facility is necessary, which could be correct depending on institutional capabilities.

Case 2 (pregnant parent with syncope): In Case 2, participants were called to a MET for a pregnant parent with syncope. The overarching goals of this simulation were to recognize that the patient was pregnant, to discuss the differential of syncope in a pregnant patient, to assess the patient and obtain available diagnostic tests, and to ultimately determine the patient's disposition. The preferable disposition decision was to evaluate and stabilize the patient at the nearest ED unless the patient’s condition warranted immediate obstetric care via advanced life support (ALS) transfer. The patient in the simulation may have asked to drive herself to an adult hospital, which should have resulted in a discussion regarding the potential risks to the patient and the unborn child.

Case 3 (parent with chest pain): This case described a middle-aged parent with sudden-onset, crushing, left-sided chest pain associated with shortness of breath. Based on history, the key steps of this simulation are for participants to recognize acute coronary syndrome and to subsequently call 911 to arrange for immediate transfer to an adult facility with catheterization capabilities, since that is the definitive treatment and time to catheterization affects prognosis. If the simulation were carried out in a general ED, the patient should be cared for in that ED rather than being transferred via ALS. Other interventions the team could offer include aspirin and obtaining an ECG while awaiting paramedic arrival. Those participants who requested that an ECG be performed were given a tracing to interpret as ST elevation myocardial infarction. Similar to the first two case scenarios, this case highlights that although adults can decline transfer and stabilizing treatment, it is important to have a conversation surrounding the benefits of transfer and the potential harms of not transferring the patient, including death, as in this patient.

Case 4 (sibling with anaphylaxis): The fourth and final case presents participants with a patient’s sibling in a hospital parking garage who was found to have sudden-onset anaphylaxis. The goals of this simulation included locating the patient’s sibling, obtaining vital signs, performing a physical exam, collecting a brief history, and stating concern for anaphylaxis. The participants were to administer the epinephrine autoinjector and recommend transfer to the ED as key management steps. Participants were expected to discuss transfer to the emergency department with the patient's guardian.

For each case scenario, participants were expected to communicate with the charge nurse and attend on-call regarding the patient disposition, including the need to transfer to the pediatric ED or outside facility for definitive care.

Debriefing

Facilitators debriefed participants using the Promoting Excellence and Reflective Learning in Simulation (PEARLS) Debriefing Framework. This flexible framework allows simulation facilitators to tailor the discussion toward the participants’ learning objectives to improve clinical decision-making and interprofessional collaboration ​​[8]. Based on the PEARLS debriefing framework, we recommended beginning the debriefing session with a discussion surrounding participants’ initial feelings and reactions, followed by participants’ description of the case scenario. The simulation facilitator could gear the discussion toward questions that arose during the case description or delve further into observations made by the learners regarding the initial MSE and clinical decision-making for outside medical emergencies. We recommended a debriefing timeframe of at least 10 to 15 minutes in a location away from where the simulation occurred.

Post-simulation Survey

Following the debrief, participants were asked to complete a post-simulation survey (Appendix B) via a QR code, which redirected survey respondents to REDCap. Participants were asked whether they agreed with the statements pertaining to the outside MET simulation using a Likert scale (1=strongly disagree, 2=disagree, 3=neutral, 4=agree, 5=strongly agree), as well as their confidence in their abilities following the simulation (1=very unconfident, 2=unconfident, 3=neutral, 4=confident, 5=very confident). These questions prompted participants to reflect on the simulation processes and the clinical decision-making involved in outside medical emergencies described in the learning objectives. The survey also included several open-ended questions to provide participants the opportunity to describe the overall impact of the simulation on their jobs and a space for participants to provide feedback on the simulation.

Results

Of the 10 voluntary surveys sent out to participants, nine post-simulation surveys were completed; PEM attendings completed five, and PEM fellows completed four. The survey results are summarized in Tables 1-2.

**Table 1:** Participants' simulation experience MET: medical emergency team

	Strongly disagree N(%)	Disagree N(%)	Neutral N(%)	Agree N(%)	Strongly agree N(%)
Statement					
The cases presented during the simulation are relevant to my work	0 (0%)	0 (0%)	0 (0%)	3 (33%)	6 (67%)
The simulation cases were realistic	0 (0%)	0 (0%)	0 (0%)	3 (33%)	6 (67%)
The simulation cases were effective in teaching basic outside medical evaluation team skills	0 (0%)	0 (0%)	0 (0%)	4 (44%)	5 (56%)
The debrief promoted reflection and team discussion	0 (0%)	0 (0%)	0 (0%)	2 (22%)	7 (78%)
The group discussion helped me develop and prioritize evaluation and management options for an outside MET response	0 (0%)	0 (0%)	0 (0%)	4 (44%)	5 (56%)
The facilitators created a safe environment for discussion and exploration	0 (0%)	0 (0%)	0 (0%)	1 (11%)	8 (89%)

**Table 2:** Participants' clinical confidence after the simulation MET: medical emergency team

Statement	Very unconfident N(%)	Unconfident N(%)	Neutral N(%)	Confident N(%)	Very confident N(%)
After participating in this session, how confident are you in your ability to					
Demonstrate ability to assess, stabilize, and triage during outside MET	0 (0%)	0 (0%)	1 (11%)	5 (56%)	3 (33%)
Formulate a list of possible diagnoses and prioritize elements of evaluation in the outside MET setting	0 (0%)	0 (0%)	1 (11%)	5 (56%)	3 (33%)
Understand outside MET team capability	0 (0%)	0 (0%)	1 (11%)	6 (67%)	2 (22%)
Construct a disposition plan after initial outside MET stabilization	0 (0%)	0 (0%)	0 (0%)	6 (67%)	3 (33%)
Utilize effective team leadership, roles, and communication strategies	0 (0%)	0 (0%)	0 (0%)	5 (56%)	4 (44%)

Participants largely found that the simulation cases were relevant to their work, and they felt confident in their ability to manage a MET response following participation in this session. Following this simulation, 67% of respondents felt that they understood the capabilities of the MET. Concerning the open-ended question about how the simulation could be improved, there were several key pieces of feedback. First, for the unarousable adult with an opioid overdose, a respiratory rate of 12 is within a normal range for an adult and would not necessarily be concerning for respiratory depression. Second, for the pregnant parent with syncope, her heart rate is technically considered normal, as pregnant patients will have relative tachycardia during pregnancy. One quick measure that can be used to assess hemodynamic stability in the pregnant patient is the shock index (heart rate divided by systolic blood pressure). Feedback regarding vital sign changes for adult and pregnant patients will be important to incorporate into the simulations to improve future sessions.

## Discussion

This simulation aimed to provide PEM trainees and attendings a safe learning environment to practice quickly assessing and determining the disposition of outside medical emergencies. The feedback from survey respondents was overwhelmingly positive, with most participants feeling as though the simulations were relevant to their work and felt realistic. Most respondents felt that the learning environment was safe and that they felt more comfortable managing an outside medical response after the simulation. The target audience included PEM fellows and attendees. However, depending on the personnel constituting a MET per institutional guidelines, this curriculum could target a broader audience, including nurses, pediatric residents, and other medical professionals. The design of the curriculum allowed participants to review the differential diagnoses for adult presentations, including altered mental status and chest pain, as well as syncope in a pregnant patient. The curriculum also allowed participants to manage a pediatric patient outside of the ED while communicating with the parent regarding the management and disposition of the child. Our cohort provided critical feedback regarding vital sign changes seen in opioid ingestion, as well as in pregnancy. It will be important to incorporate this feedback for future iterations of this simulation. The curriculum also highlighted the importance of stabilizing a patient in one’s own ED and alerting the ED staff versus deciding when an emergent transfer is needed for definitive care and how to carry out the transfer process. Depending on where the simulation is carried out (pediatric vs. general ED), transferring the patient to the respective ED may have been appropriate instead of emergently transferring to another hospital for definitive care. In future simulation iterations, it will be important to alert participants that numerous patient dispositions are possible and depend on their respective ED’s capabilities.

Limitations inherently exist when utilizing the Likert scale as the main assessment form. The Likert scale is susceptible to response bias, including agreement or acquiescence bias [9]. One way of mitigating agreement bias is to write more open-ended questions [10]. Additionally, assessing participants’ opinions does not necessarily correlate with true proficiency and understanding of the MET response. To better understand participants’ knowledge prior to the simulation and their understanding of the MET response after participating, it would be helpful to administer pre- and post-simulation surveys. Another limitation includes our small sample size, with only two sites participating. Finally, another limitation includes the variability between institutions, such as the variability in personnel and equipment.

## Conclusions

MET responses are infrequent but high-stress situations that require prompt medical decision-making. This simulation curriculum directly impacts PEM providers' preparedness for outside adult and pediatric medical emergencies. In future iterations of this simulation, we aim to incorporate previous participants' feedback, such as having both a pre- and post-simulation survey and including more open-ended questions to mitigate agreement bias, among others. Expanding this simulation curriculum and future research will be important to ensure better preparedness for non-hospitalized emergencies across institutions.

## Appendices

Appendix A

Guide for Simulation Facilitators

Case 1: Unresponsive adult overdose

Summary: A 42-year-old unarousable parent is in the waiting room of an outpatient clinic

Total time: 10 min max

Learning objectives

1. Locate the position of the non-hospitalized person experiencing an emergency (located at outpatient clinic for this scenario)

5. 2. Evaluate the patient by performing

    a. Exam

        i. Basic vitals: BP: 90/50, HR: 60, O2Sat: 92%, RR: 12, T: 37, if requested, glucose is 80

        ii. Physical exam: Somnolent, pinpoint pupils but otherwise non-focal exam

    b. Brief history

3. Recognize altered mental status (AMS), concern for opioid overdose

4. Verbalize a differential diagnosis for AMS in an adult

5. Recommend diagnostics, including

    a. POCT glucose (POCT glucose = 80 if asked for glucometer from clinic)

    b. ECG using ECG machine from clinic (ECG shows normal sinus rhythm)

    c. Manage opioid overdose

    d. Support ABC: Provide airway support as needed

    e. Administer naloxone

    f. Determine patient disposition

6. Recommend transfer to the ED for observation/evaluation OR if reasons for further concern call 911

7. Alert the primary attending in the ED and verbalize the need for transfer

Scenario

1. Introduction: A 42-year-old parent of a 6-year-old patient with type 1 diabetes presents with AMS and inability to be aroused

2. Brief history obtained from parent’s child

    a. Signs/symptoms: The medical assistant came to the patient's room and could not arouse the parent. More than two strong pulses and breathing spontaneously

    b. Allergies: Unknown

    c. Medications: Unknown; the kid states that the parent “takes some pills”

    d. Past medical history: Unknown; when the parent awakens, the parent states they are on methadone and in recovery for heroin addiction

    e. Last meal: Unknown; kid states that parent last ate during breakfast

    f. Events leading up to the current scenario: Arrived at the clinic, checked in, and then sat and fell asleep waiting for your appointment

3. Following administration of naloxone

    a. Patient awakens and is combative and appears intoxicated. We want them to understand that while this is a patient we *could* assess in our ED, there is an agreement that we cannot transport them back across the street ourselves and will need to call ALS to transport them to our ED

Supplemental material: None

Debrief points

1. Discuss differential diagnosis for AMS in an adult

    a. Hypoglycemia

    b. Electrolyte abnormalities

    c. Intoxication

    d. Opioid overdose

    e. Arrhythmia

    f. Hypovolemia

    g. Other

2. Discuss the need for transfer to stabilize the patient while also considering that the patient is an adult and can decline the recommendations for transfer

3. Review how the group functioned as a team

Case 2: Pregnant parent with syncope

Summary: A 33-year-old pregnant parent has a syncopal episode en route to a maternal fetal medicine appointment in an outpatient clinic adjacent to the hospital

Total time: 10 min max

Learning objectives

1. Locate the position of the pregnant parent with syncope (hospital garage for this scenario)

2. Evaluate patient

3. Exam

    a. Basic vitals: BP: 108/75, HR: 110, O2Sat: 98%, RR: 18, T: 37C

4. Brief history

5. Recognize and verbally confirm pregnancy, as well as gestational age, with patient

6. Verbalize a differential diagnosis for syncope in pregnancy

7. Recommended diagnostics

    a. POCT glucose (glucose = 80 if asked for from clinic)

    b. ECG using clinic ECG machine (normal sinus rhythm)

    c. Determine disposition of patient

        i. Transfer to the ED for observation/evaluation

        ii. If reasons for further concern are present, call 911 for transfer via ALS for definitive care at a facility with obstetric services

        iii. Patient can request to drive herself to the preferred hospital

        iv. Alert primary attending in the ED and verbalize the need for transfer

Scenario

1. Introduction: A 33-year-old pregnant parent with syncope while walking to an appointment this morning in the hospital garage

2. Brief history

    a. Signs/symptoms: Patient was walking, felt dizzy, and sat down. She subsequently had loss of consciousness (LOC)

    b. Allergies: Penicillin

    c. Medications: Prenatal vitamin daily

    d. Past medical history: She is about 20 weeks pregnant. This is her first pregnancy. She had gallbladder removal a few years ago

    e. Last meal: Had an egg sandwich and coffee about 15 minutes prior to arrival at the hospital

    f. Events leading up to scenario: Patient had just been dropped off by her partner and was walking to the clinic when she suddenly felt dizzy/woozy. So, she sat down and then had a brief LOC. She sustained no trauma. She denies shortness of breath (SOB)

    g. After patient evaluation: She is cooperative and requests to drive per her own vehicle (POV) with her partner to the preferred hospital and to call her OB

Supplemental material: None

Debrief points

1. Discuss differential diagnosis for syncope in pregnancy

    a. Embolus

    b. Orthostasis

    c. Hypovolemia

    d. Hypoglycemia

    e.Arrhythmia

    f. Other

2. Discuss the need for transfer while realizing that the patient is an adult and can decline recommendations

3. Call out team dynamics

Case 3: Adult chest pain

Summary: Adult parent with sudden onset chest pain concerning for acute chest syndrome (myocardial infarction) at the hospital garden space

Total time: 10 min max

Learning objectives

1. Locate patient

2. Evaluate patient

3. Exam

    a. VS: BP: 145/90 HR: 90, RR: 20, O2sat: 96% RA, T: 37

    b. If in a clinic setting and a POCT glucose monitor is available, can request (POCT glucose = 120)

    c. Brief history

    d. Verbalize concern for myocardial infarction

    e. Recommend interventions such as aspirin, electrocardiogram (ECG) in the ED (if this case is in the clinic setting, can ask for ECG machine), transfer by calling 911

    f. Determine disposition

    g. Team to discuss with primary attending in the ED and verbalize the need for transfer given that time to catheterization is crucial

    h. Team to engage in discussion with patient regarding the need for further evaluation by medics and transfer to adult hospital

Scenario

1. Introduction: A 38-year-old parent with sudden-onset severe crushing pain in the left chest radiating to the left arm. The parent is bringing the daughter to an appointment when the parent developed this sudden 8/10 pain. The parent is having some SOB associated with the pain. The parent also has dyspnea upon exertion

2. Brief history

    a. Signs/symptoms: Sudden onset left-sided chest pain

    b. Allergies: None

    c. Medications: Multivitamin daily, Zantac

    d. Past medical history: Anxiety, GERD

    e. Last meal: Grabbed a Big Mac on patient’s way up to the hospital garden

    f. Events leading up to current scenario: Seven-year-old daughter is admitted for fever, and parent came up to the hospital garden for some air and parent suddenly developed this left-sided chest pain

    g. Family history: If asked, the parent’s uncle passed away from a heart attack when he was middle aged

3. The parent initially declines evaluation, medications, and transport via ALS to the nearest adult hospital, stating that they’d like to ride it out and don’t want to pay for a medical exam or ambulance. The team eventually convinced the parent to take baby aspirin, obtain ECG in the ED/via medics, and allow the team to transfer the parent to the nearest adult hospital

Optional supplemental material: ECG-Anterior-STEMI-Evolving-2-1024x497.jpg (1024×497) (litfl.com)

Debrief points

1. Highlight the importance of calling 911 for acute chest syndrome as time to catheterization lab matters and this is the fastest way to mobilize the patient

2. Discuss the case with the adult ED attending for emergent medic transfer and call the medical transfer center to initiate transfer

3. Discuss the process of how the patient was convinced to be transferred

4. Call out team dynamics

Case 4: Anaphylaxis in the sibling of the patient

Summary: A Sibling of a patient with sudden onset anaphylaxis in the hospital garage. Consider using a low-fidelity mannequin for the scenario

Total time: 10 min max

Learning objectives

1. Locate the position of the patient (outpatient clinic)

2. Evaluate mannikin

3. Exam

    a. Basic vitals:  BP: 90/45 HR: 130, RR: 35, O2sat: 95% RA, T: 37, if POCT glucose requested glucose = 120

    b. Brief histor

4. Verbalize concern for anaphylaxis

5. Recommend interventions such as ean pinephrine autoinjector

6. Administer epinephrine autoinjector

7. Recommend transfer to the pediatric ED

8. Alert the primary attending in the ED and verbalize the need for transfer

9. Team to keep parent updated

Scenario

1. Introduction: A four-year-old child with sudden onset urticaria and wheezing. After the sibling was evaluated for a nursemaid, the patient walked to the car with the family. The patient was walking to the car with no apparent trigger. The patient has a severe tree nut allergy

2. Brief history

    a. Signs/symptoms: Sudden onset of urticaria and wheezing

    b. Allergies: Tree nuts

    c. Medications: Multivitamin daily

    d. Past medical history: tree nut allergy; has never required an epinephrine autoinjector. The patient developed hives once when they had peanut butter as an infant and has since been confirmed to have a severe allergy. The patient has an epinephrine autoinjector, but it’s at home

    e. Last meal: Had a Starbucks cookie about 30 minutes prior to symptom onset and was told that the cookie was nut free

    f. Events leading up to scenario: Patient was calmly watching a show on a tablet when mother noticed difficulty breathing and rash while waiting for sibling to be called back

3. Following epinephrine autoinjector administration, the parent cooperates and agrees with ED transfer

Supplemental material: None

Debrief points

1. The epinephrine autoinjector is in the medical emergency team (MET) backpack.  If not found, ask clinic staff if they have diphenhydramine/cetirizine or epinephrine available to administer

2. Discussion about alerting ED about transfer

3. Call out team dynamics

Appendix B

Outside Medical Emergency Team (MET) PEM Fellows Simulation Evaluation

Case Presented:  Outside medical emergency team

Instructor(s):   _______  (free text)

Date:  _______

Institution: __________

Year of PEM fellowship: (1st, 2nd, 3rd)

**Table 3:** Survey of participants' simulation experience MET: medical emergency team

Statement	Strongly disagree	Disagree	Neutral	Agree	Strongly agree
The cases presented during the simulation are relevant to my work.	1	2	3	4	5
The simulation cases were realistic.	1	2	3	4	5
These simulation cases were effective in teaching basic outside medical evaluation team skills.	1	2	3	4	5
The debrief promoted reflection and team discussion.	1	2	3	4	5
The group discussion helped me develop and prioritize evaluation and management options for an outside MET response.	1	2	3	4	5
The facilitators created a safe environment for discussion and exploration.	1	2	3	4	5

**Table 4:** Survey of respondents' clinical confidence after the simulation MET: medical emergency team

After participating in this session, how confident are you in your ability to	Very unconfident	Unconfident	Neutral	Confident	Very confident
Demonstrate ability to assess, stabilize, and triage during outside MET	1	2	3	4	5
Formulate a list of possible diagnoses and prioritize elements of evaluation in the outside MET setting	1	2	3	4	5
Understand outside MET team capability	1	2	3	4	5
Construct a disposition plan after initial outside MET stabilization	1	2	3	4	5
Utilize effective team leadership, roles, and communication strategies	1	2	3	4	5

Open-Ended Survey Questions

1.  Can you list/describe one or more ways this session will change how you do your job?

2. How could we improve this simulation?

3. Additional comments:
